# Deviated binding of anti-HBV nucleoside analog *E*-CFCP-TP to the reverse transcriptase active site attenuates the effect of drug-resistant mutations

**DOI:** 10.1038/s41598-024-66505-z

**Published:** 2024-07-08

**Authors:** Yoshiaki Yasutake, Shin-ichiro Hattori, Hiroki Kumamoto, Noriko Tamura, Kenji Maeda, Hiroaki Mitsuya

**Affiliations:** 1 Bioproduction Research Institute, National Institute of Advanced Industrial Science and Technology (AIST), Sapporo, 062-8517 Japan; 2 Computational Bio Big-Data Open Innovation Laboratory (CBBD-OIL), AIST, Tokyo, 169-8555 Japan; 3 National Center for Global Health and Medicine (NCGM) Research Institute, Tokyo, 162-8655 Japan; 4 Department of Pharmaceutical Sciences, Nihon Pharmaceutical University, Saitama, 362-0806 Japan; 5 Division of Antiviral Therapy, Joint Research Center for Human Retrovirus Infection, Kagoshima University, Kagoshima, 890-8544 Japan; 6 Experimental Retrovirology Section, HIV and AIDS Malignancy Branch, National Cancer Institute, National Institutes of Health, Bethesda, MD 20892 USA; 7 Department of Clinical Sciences, Kumamoto University Hospital, Kumamoto, 860-8556 Japan

**Keywords:** X-ray crystallography, Antiviral agents, Hepatitis B virus, Antimicrobial resistance

## Abstract

While certain human hepatitis B virus-targeting nucleoside analogs (NAs) serve as crucial anti-HBV drugs, HBV yet remains to be a major global health threat. *E*-CFCP is a 4′-modified and fluoromethylenated NA that exhibits potent antiviral activity against both wild-type and drug-resistant HBVs but less potent against human immunodeficiency virus type-1 (HIV-1). Here, we show that HIV-1 with HBV-associated amino acid substitutions introduced into the RT’s dNTP-binding site (N-site) is highly susceptible to *E*-CFCP. We determined the X-ray structures of HBV-associated HIV-1 RT mutants complexed with DNA:*E*-CFCP-triphosphate (*E*-CFCP-TP). The structures revealed that exocyclic fluoromethylene pushes the Met184 sidechain backward, and the resultant enlarged hydrophobic pocket accommodates both the fluoromethylene and 4′-cyano moiety of *E*-CFCP. Structural comparison with the DNA:dGTP/entecavir-triphosphate complex also indicated that the cyclopentene moiety of the bound *E*-CFCP-TP is slightly skewed and deviated. This positioning partly corresponds to that of the bound dNTP observed in the HIV-1 RT mutant with drug-resistant mutations F160M/M184V, resulting in the attenuation of the structural effects of F160M/M184V substitutions. These results expand our knowledge of the interactions between NAs and the RT N-site and should help further design antiviral NAs against both HIV-1 and HBV.

## Introduction

Hepatitis B virus (HBV) persistently infects approximately 300 million people worldwide, and approximately 1 million people die annually from liver cancer, cirrhosis, and other diseases caused by HBV infection^1–3^. Nucleoside analogs (NAs) are crucial anti-HBV drugs that target the reverse transcriptase (RT) dNTP-binding site (N-site) of HBV polymerase (Pol), but the emergence of drug-resistant viruses during the required continuous drug administration represents a major problem^4–6^. Particularly, common amino acid substitutions (e.g., L180M/M204V in HBV RT) render the two major anti-HBV drugs, entecavir (ETV) and lamivudine (3TC), virtually ineffective^5^. However, a recently approved anti-HBV NA, tenofovir alafenamide (TAF), is potent against such drug-resistant viruses, and no significant amino acid substitutions conferring TAF resistance have been identified to date^7,8^. Therefore, TAF now serves as a first choice NA for anti-HBV treatment, while the TAF’s prototypic tenofovir has the well-known risks of causing renal and bone-related adverse events due to its toxicity^9^. Moreover, all the currently available anti-HBV drugs require the QD (once daily) dosing regimen, which may hamper the quality of life (QOL) in patients with chronic hepatitis. Thus, the development of new drugs that are highly effective against both wild-type and drug-resistant viruses and allow better dosing schedules is urgent.

**Figure 1:**
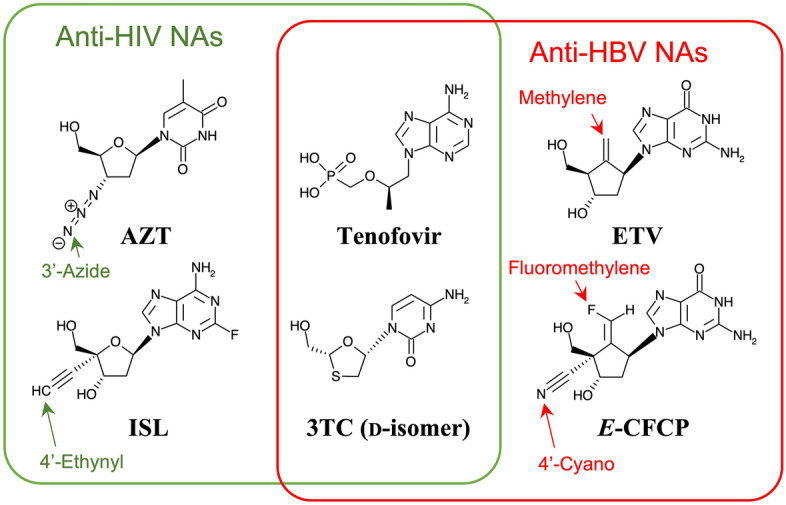
Typical anti-HIV-1 and anti-HBV NAs featured in this study. Modification group characteristics of each compound are indicated by arrows. Tenofovir and 3TC with both anti-HIV-1/HBV activity have no protruding modifications, whereas the anti-HBV- or anti-HIV-1-specific compounds are bulkier with protruding modifications and may thus exhibit specificity for the respective HBV/HIV-1 RT N-sites.

We recently reported a novel anti-HBV NA, *E*-CFCP, with high potency against both wild-type and drug-resistant HBV^10,11^. Of note, *E*-CFCP and its triphosphate form (*E*-CFCP-TP) are chemically stable in the human body and thus exhibit remarkable long-acting properties. Moreover, *E*-CFCP is less toxic than ETV and TAF. These attractive properties could allow a once-weekly dosing regimen that is advantageous in improving the QOL of infected patients^10^. *E*-CFCP is a bulky guanosine analog with a unique exocyclic fluoromethylene and 4′-cyano group (Fig. 1). Understanding the structural mechanism responsible for the potent anti-HBV activity of *E*-CFCP is important; thus, structural study of HBV Pol complexed with *E*-CFCP-TP by X-ray crystallography is essential.

HBV Pol is a 90-kDa multifunctional protein comprising four distinct domains: terminal protein domain (TP), flexible spacer, RT, and RNase H domain (RH). HBV Pol is a unique protein in that it serves as a protein primer with a Tyr residue (Tyr63) in the TP domain prior to successive DNA chain synthesis within the nucleocapsid core particle^12,13^. Unfortunately, HBV Pol is notorious for being extremely unstable and insoluble, and obtaining large amounts of soluble HBV Pol suitable for structural study using any expression hosts or cell-free systems has not been achieved to date^14^. Two factors are potentially responsible for the undesirable characteristics of HBV Pol. First, HBV Pol has been known to interact with various cellular proteins. For example, purified HBV Pol from HEK293 cells showed co-purification with molecular chaperones such as Hsp90, Hsc70, Hsp60, and host factor DDX3^15^. Similarly, the molecular chaperone GroEL has been detected in the purified HBV Pol fragments produced by *Escherichia coli*^16^. In addition, a recent study reported that additional interactions with host factors such as RBM38 are required for correct incorporation into core particles accompanied by pregenomic RNA (pgRNA)^17^. Catalytically active HBV Pol is considered to exist only in core particles together with pgRNA, and cell-imaging analysis has suggested that most HBV Pol molecules outside the core particle are transported to mitochondria anchored by Hsp60^18^. These findings strongly suggest that HBV Pol is highly flexible, adhering, in multiple transient states, to various host factors. Second, the HBV genome is very compact, and the genes encoding core, surface antigen, and X proteins partially or fully overlap with the Pol gene with a reading-frame shift^19^. Thus, the HBV Pol amino acid sequence might be severely biased by the sequences of these overlapped proteins^20,21^, which might also be related to the unstable and insoluble properties of recombinant HBV Pol. Previous studies have shown that a small amount of full-length HBV Pol and each TP/RT/RH domain could be successfully purified in the presence of molecular chaperons, detergents, or chemical solubilizers, and the priming activities could be detected by measuring the incorporation of radioisotope-labeled dNTPs to Tyr63 in TP^14,16^. We recently reported the detection of RT elongation products with partially purified HBV RT/RH using FITC-5′-labeled DNA primers^22^. In any case, the stability and solubility of HBV TP/RT/RH are extremely limited, thereby hindering structural study by X-ray crystallography and cryo-electron microscopy.

The N-site structure of HBV Pol, which is essential for understanding NA binding and resistant mechanisms, has long been elucidated using in silico structural models based on the crystal structures of human immunodeficiency virus type-1 (HIV-1) RT^10,23^. The structure of the N-sites of HIV-1 RT and HBV Pol are considered to be quite similar, as the amino acid sequences that form the N-sites (referred to as motifs A, B, C, D, and E) are moderately conserved in both (Supplementary Fig. S1a)^23,24^. In fact, some NAs are potent against both HBV and HIV-1, while the anti-HBV agents ETV and *E*-CFCP are only moderately active against HIV-1^10,11^. Very recently, the structural prediction of full-length HBV Pol using AlphaFold2^25^ has been reported, demonstrating the similarity of the N-site structure between HIV-1 RT and HBV Pol^26^. In contrast to these computational approaches, we have experimentally constructed HIV-1 RT mutants with HBV-associated amino acid substitutions at the N-site (Table 1). Interestingly, some of the HBV-associated mutations (such as L74V, Y115F and Q151M) are already known as drug-resistant mutations in HIV-1 RT. We found that the three amino acid substitutions Y115F/F116Y/Q151M (3MB) in the HIV-1 RT N-site render HIV-1 highly sensitive to anti-HBV drugs ETV and 3TC. Furthermore, the mutations F160M/M184V, which correspond to the reported drug-resistant L180M/M204V in HBV RT, simulated drug resistance against ETV/3TC in HIV-1^3MB^^22,27,28^. A series of antiviral and experimental structural studies of HIV-1 RT^3MB^ and RT^3MB/M184V/F160M^ revealed a key determinant for ETV/3TC sensitivity (Met151), an atypical binding mode of ETV-TP/3TC-TP to the N-site involving pushing of the Met184 sidechain, and a bulging structure of the N-site due to F160M. All of these findings provide a reasonable explanation for the mechanism of ETV/3TC sensitivity and resistance in HBV^23,29,30^. It should also be noted that these structural properties have not been predicted by previous in silico modeling studies. Thus, we presume that this artificial system is valuable for understanding other NA-binding and resistant mechanisms of HBV.

**Table 1:** List of HIV-1 mutants with HBV-mimicking mutations in RT.

Identifier	Amino acid substitutions in HIV-1 RT N-site				Viral replication	References
	β2–β3	Motif A	Motif B	Motif C		
HIV^WT^					++	–
HIV^1M^			Q151M		++	^27^
HIV^2M^			Q151M/F160L		–	^27^
HIV^3MA^		G112S/D113A	Q151M		+	^27^
HIV^3MB^		Y115F/F116Y	Q151M		++	^27^
HIV^3MC^	I63V/L74V		Q151M		++	^27^
HIV^3MB/M184V^		Y115F/F116Y	Q151M	M184V	++	^22,28^
HIV^3MB/F160M/M184V^		Y115F/F116Y	Q151M/F160M	M184V	++	^22,28^
HIV^3MB/F160M/Q182G/M184V^		Y115F/F116Y	Q151M/F160M	Q182G/M184V	–	^28^
HIV^5M^		G112S/D113A/Y115F/F116Y	Q151M		N.T.	Unpublished
HIV^6M^		G112S/D113A/Y115F/F116Y	Q151M/F160L		–	^31^
HIV^7MA^		G112S/D113A/Y115F/F116Y/V118L	Q151M/F160L		–	^31^
HIV^7MB^		G112S/D113A/Y115F/F116Y	L149I/Q151M/F160L		–	^31^
HIV^7MC^		G112S/D113A/Y115F/F116Y	Q151M/I159L/L160L		–	^31^
HIV^5MA^		Y115F/F116Y	Q151M/I159L/F160L		–	Unpublished
HIV^6MA^		Y115F/F116Y	L149I/Q151M/I159L/F160L		–	Unpublished
HIV^7MD^		Y115F/F116Y/V118L	L149I/Q151M/I159L/F160L		–	Unpublished
HIV^4M^	L74V	Y115F/F116Y	Q151M		–	This study
HIV^4MA^	I63V	Y115F/F116Y	Q151M		–	This study
HIV^5MB^	I63V/L74V	Y115F/F116Y	Q151M		++	This study

F160M, Q182G, and M184V in HIV-1 RT correspond to the drug-resistant L180M, S202G, and M204V in HBV RT, respectively. L74V, Y115F, Q151M, and M184V have also been reported as drug-resistant mutations in HIV-1.

N.T., not tested.

The present study aimed to elucidate the mechanism of potent antiviral activity of *E*-CFCP against wild-type and drug-resistant HBV using HIV-1 with HBV-associated mutations in RT. We first examined the antiviral activity of typical anti-HBV NAs, including *E*-CFCP, against HIV-1 with HBV-associated and drug-resistant mutations in RT. We also determined the crystal structures of HIV-1 RT^3MB^ and RT^3MB/L74V (4M)^ in complex with DNA and *E*-CFCP-TP. The electron density map of exocyclic fluoromethylene and the 4′-cyano group showed that *E*-CFCP-TP is slightly skewed but reliably bound to the N-site. These X-ray structural models also rationalize how *E*-CFCP-TP escapes from the structural effects of drug-resistant mutations.

## Results and discussion

### Design of HIV-1 RT mutants that mimic the HBV RT N-site

Five amino acid sequence motifs (motifs A–E) give rise to the N-site of HIV-1 RT (Supplementary Fig. S1a). In our previous studies, we examined various combinations of amino acid substitutions in motifs A–E and found that the HBV-RT-associated 3MB mutant comprising Q151M in motif B and Y115F/F116Y in motif A is useful as a surrogate for structurally studying HBV RT since HIV-1 harboring RT^3MB^ turned out to be highly sensitive against typical anti-HBV NAs, ETV, and 3TC (Table 1)^22,27,28,31^. In this study, to further mimic HBV-RT, we were also interested in the HBV-associated L74V/I63V mutation residues in the β2–β3 region (Supplementary Fig. S1b). Leu74 and Ile63 serve to stabilize the base moiety of the primer nucleotide base-paired with NAs/dNTP at the N-site through stacking interactions. Thus, the substitution of these residues with Val, which is smaller, possibly affects the binding position of the bound dNTP/NAs. Here, we constructed three additional mutants (3MB + L74V (4M), 3MB + I63V (4MA), and 3MB + L74V/I63V (5MB); Table 1) and evaluated their viral replicability, *E*-CFCP susceptibility, and enzyme activity relative to previously constructed mutants (1M, 3MB, 3MB/M184V, and 3MB/F160M/M184V).

### Virus replication kinetics, RT activity, and NA susceptibility

Wild-type HIV-1 (HIV-1^WT^) and seven HIV-1 variants harboring RT mutations were used, and their replication was examined. Their replication kinetics indicated that although HIV-1^4M^ and HIV-1^4MA^ completely failed to replicate when cultured for up to 7 days, the other five variants propagated similarly to or better than HIV-1^WT^ (Supplementary Fig. S2a). HIV-1 variants 4M and 4MA were considered able to propagate, but their replication capacity was completely lost. As shown in Supplementary Fig. S2b, the magnitude of enzymatic activity of RT^4M^ and RT^4MA^ was slightly lower than that of RT^WT^, and further investigation is needed to explain the difference between HIV-1 RT activity and the propagation profile of HIV-1 with these mutations. In contrast, although the proliferation of HIV-1^3MB^ was greater than that of HIV-1^WT^ and the virus level at day 7 was more than twofold higher, the enzyme activity of RT^3MB^ was lower than that of RT^WT^ and comparable to that of RT^4M^ or RT^4MA^. Taken together, these results suggested that either the L74V or I63V mutations in RT were highly detrimental to viral replication. However, the presence of both I63V and L74V mutations improved viral replication fitness compared with the presence of single mutations. The results also indicated that there is no perfect correlation between viral propagation and the activity of the purified enzyme. Additionally, the I63V/L74V mutations do not have any adverse effects on RT enzyme activity. It is probable that factors other than the levels of RT enzyme activity can influence viral replicability.

We selected five replicable variants (HIV-1^1M^, HIV-1^3MB^, HIV-1^3MB/M184V^, HIV-1^3MB/L160M/M184V^, and HIV-1^5MB^) for an antiviral assay to investigate their sensitivity against typical anti-HBV/HIV-1 NAs (Fig. 1), including ETV (anti-HBV), *E*-CFCP (anti-HBV), azidothymidine (AZT, anti-HIV), islatravir (ISL, formerly EFdA, anti-HIV), 3TC (anti-HIV/HBV), and TAF (anti-HIV/HBV). As shown in Table 2, all NAs showed antiviral activity against HIV-1^WT^; ISL was the most potent (IC_50_: 0.61 nM), followed by AZT (9.1 nM), TAF (53.8 nM), and 3TC (282 nM). HBV-specific NAs, ETV and *E*-CFCP, were weakly active against HIV-1^WT^ (1,781 and 519 nM, respectively). ETV had the lowest activity against HIV-1^WT^ among the tested compounds. Next, we examined the changes in the NA sensitivity of HIV-1 harboring HBV-associated and drug-resistant mutations, and the results for AZT/ISL/TAF/ETV/3TC were comparable to those of previous studies^27,28^. In addition, we showed that sensitivity to *E*-CFCP changes in a trend similar to that of ETV, i.e., susceptibility to *E*-CFCP is greatly enhanced by the Q151M mutation (1M) and further enhanced by Y115F/F116Y mutations (3MB). The results suggest that Met151 is a key determinant for *E*-CFCP binding to the N-site, as is the case for ETV^27^. Compared with HIV-1^3MB^, HIV-1^5MB^ had up to approximately 40-fold lower ETV/3TC susceptibility, consistent with a previous study on the combination of 1M and I63V/L74V^27^. Taken together, the I63V/L74V mutations in 5MB do not affect susceptibility to anti-HIV-specific NAs but reduce susceptibility to ETV/3TC. Notably, HIV-1^5MB^ maintains high sensitivity to *E*-CFCP, suggesting that the structural effects of the I63V/L74V mutation might be more compatible with *E*-CFCP binding to the N-site, as discussed later. We also confirmed that the IC_50_ value of *E*-CFCP for the drug-resistant HIV-1^3MB/F160M/M184V^ is comparable to that of TAF (Table 2), consistent with the fact that both *E*-CFCP and TAF are potent against drug-resistant HBV with L180M/M204V mutations.

**Table 2:** Results of the antiviral assay using typical anti-HBV/HIV-1 NAs.

Mutants	IC_50_ ± SD (nM)					
	AZT	ISL	TAF	3TC	ETV	*E*-CFCP
HIV-1^WT^	9.1 ± 0.4	0.61 ± 0.012	53.8 ± 4.5	282 ± 5.0	1781 ± 26	519 ± 3.3
HIV-1^1M^	43 ± 0.3 (4.7)	0.15 ± 0.0015 (0.25)	91.8 ± 5.0 (1.7)	123 ± 16 (0.44)	54 ± 2.5 (0.030)	28.3 ± 0.5 (0.055)
HIV-1^3MB^	291 ± 29 (32)	0.10 ± 0.016 (0.16)	36.2 ± 2.7 (0.67)	97.3 ± 23 (0.35)	35 ± 1.9 (0.020)	13.7 ± 0.7 (0.026)
HIV-1^3MB/M184V^	929 ± 110 (102)	3.6 ± 0.6 (5.9)	52.0 ± 1.9 (0.97)	682 ± 50 (2.4)	541 ± 135 (0.30)	158 ± 14 (0.30)
HIV-1^3MB/F160M/M184V^	364 ± 6 (40)	6.2 ± 0.1 (10)	70.0 ± 5.0 (1.3)	1033 ± 154 (3.7)	1189 ± 114 (0.67)	99 ± 11 (0.19)
HIV-1^5MB^	247 ± 1.2 (27)	0.21 ± 0.0045 (0.34)	667 ± 75 (12)	3558 ± 285 (13)	323 ± 19 (0.18)	5.9 ± 0.14 (0.011)

Values in parentheses indicate the fold-change of IC_50_ values compared with HIV-1^WT^.

### Structural analysis of HIV-1 RT^3MB^ and RT^4M^ complexed with DNA and *E*-CFCP-TP/ETV-TP/dGTP

In parallel with conducting the antiviral and enzyme assays, we also attempted to crystallize the series of HIV-1 RT mutants (RT^3MB/4M/4MA/5MB^) complexed with DNA and *E*-CFCP-TP. As a result, the structures of HIV-1 RT^3MB^ and -RT^4M^ complexed with DNA:*E*-CFCP-TP were determined at resolutions of 2.62 and 2.70 Å, respectively. We also determined the structures of HIV-1 RT^4M^ complexed with DNA:ETV-TP/dGTP at 2.32 and 2.30 Å resolutions, respectively, as reference models for comparison. The structure of HIV-1 RT^3MB^ with DNA:ETV-TP/dGTP has been previously reported^28^. Unfortunately, HIV-1^4M^ was unable to replicate, which prevented us from testing its drug susceptibility. However, we confirmed that the RT^4M^ maintained sufficient enzyme activity (Supplementary Fig. S2b). We thus used the primer-extension assay to examine and compare the susceptibility of RT^WT^, RT^3MB^, and RT^4M^ against *E*-CFCP-TP (Supplementary Fig. S3). The results confirmed that RT^4M^ is more susceptible to *E*-CFCP-TP compared to RT^WT^. We anticipated that comparing the structures of RT^3MB^ and RT^4M^ would help us understand the structural impact of L74V on the N-site, enabling us to predict the compatibility of *E*-CFCP-TP binding to the HBV RT N-site.

**Figure 2:**
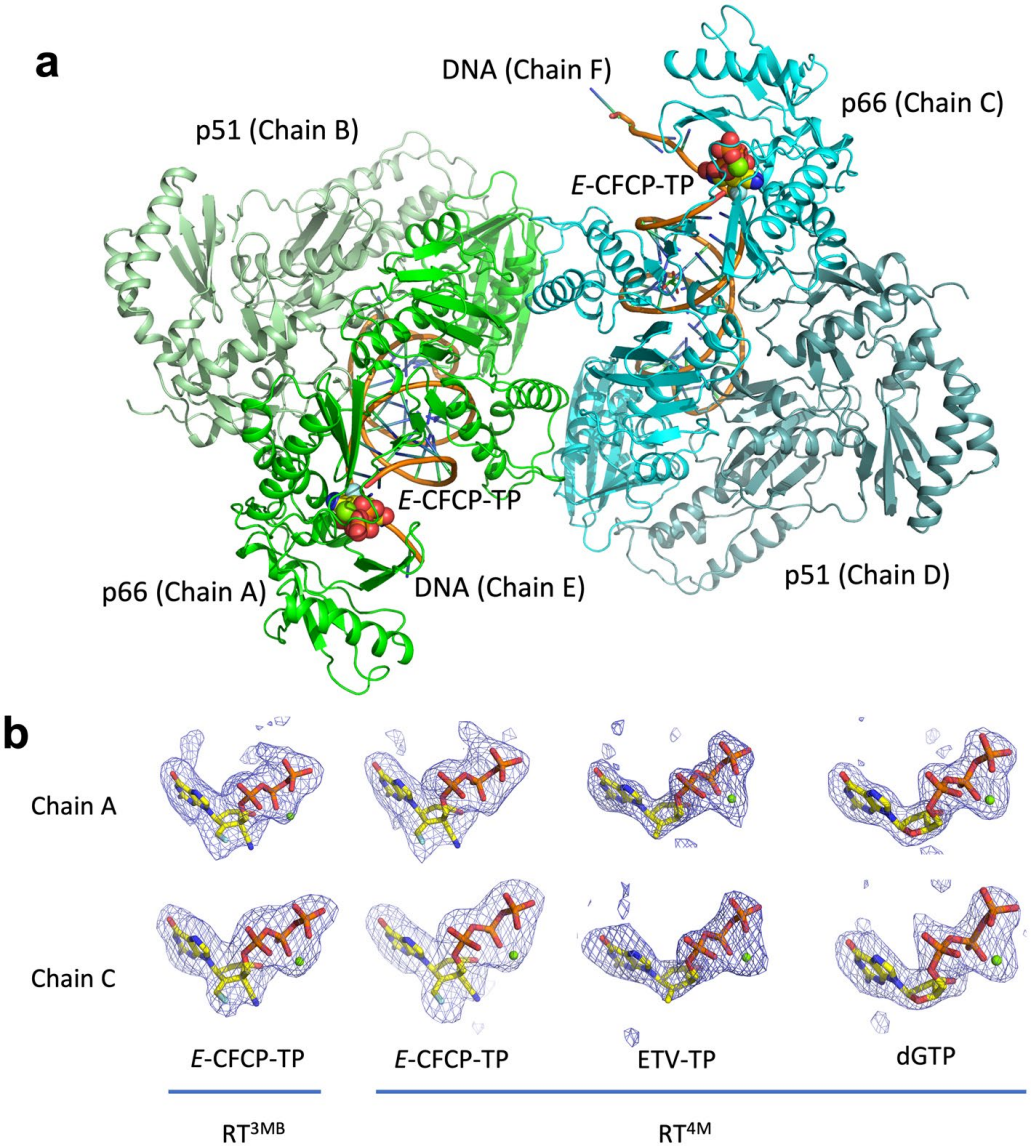
Structural analyses of the HIV-1 RT^3MB/4M^:DNA:*E*-CFCP-TP/ETV-TP/dGTP ternary complex. (**a**) Overall structure of the HIV-1 RT^4M^:DNA:*E*-CFCP-TP complex. Two HIV-1 RT^4M^ heterodimers (p66-p51) complexed with DNA and *E*-CFCP-TP in the crystallographic asymmetric unit are shown: one complex (chains A, B, and E) is colored green, and the other (chains C, D, and F) in cyan. (**b**) Simulated annealing mFo–DFc omit map for the bound *E*-CFCP-TP, ETV-TP, dGTP, and Mg^2+^ reported in this study. The maps were drawn contoured at the 2.5σ level.

The asymmetric unit of RT^3MB/4M^ crystals contains two HIV-1 RT p66-p51 heterodimers complexed with DNA (chains ABE and CDF). Chains A and C, B and D, and E and F correspond to the p66 and p51 subunits and DNA, respectively (Fig. 2a). The RT heterodimeric structures reported in the present study superimpose well with each other with a main-chain root mean square deviation (RMSD) of ~ 1.0 Å, and can also be superimposed on the previously reported HIV-1 RT structures complexed with DNA and various NAs/dNTP in closed conformation^32–37^ with a main-chain RMSD of ~ 1.2 Å. A well-defined electron density was observed for the p66-p51:DNA complex except for the N- and C-termini of p66 and p51, the internal loop of the p51 subunit (213–230), and a couple of nucleotides on the 5′-end side of the DNA. The electron density of all the amino acid sidechains creating the N-site and bound *E*-CFCP-TP/ETV-TP/dGTP was unambiguous (Fig. 2b), allowing a detailed discussion of the differences in N-site structures and *E*-CFCP-TP binding modes relative to the previously reported ETV-TP/ISL-TP/dNTP complexes of RT^WT^ and RT^3MB^^23,27,28,32–37^.

### *E*-CFCP-TP fluoromethylene and 4′-cyano exploit the expanded hydrophobic pocket at the HIV-1 RT^3MB/4M^ N-site

The clear electron density map enabled us to construct a complete atomic model of the bound *E*-CFCP-TP at the HIV-1 RT^3MB/4M^ N-site in both chains A and C. Nevertheless, the average *B*-values of the final refined *E*-CFCP-TP model were relatively high (100–110 Å^2^) compared with those of polypeptide chains and DNA close to the N-site (~ 75 Å^2^) (Table 3). In addition, the superimposition of the four chains (chain A and C of RT^3MB/4M^) indicated that the bound *E*-CFCP-TP varied in conformation with an RMSD of ~ 0.5 Å, and the electron density peak for Mg^2+^ was absent in chain A of RT^4M^ (Fig. 2b). These observations suggest that the bound *E*-CFCP-TP is slightly perturbed within the N-site of crystallized RT^3MB/4M^. In contrast, the bound ETV-TP/dGTP are well ordered and superimposed with each other with an RMSD of 0.2–0.3 Å, and their average *B*-values were similar to those of polypeptide chains and DNA (~ 60 Å^2^), suggesting that the bound ETV-TP/dGTP is rather static within the N-site (Supplementary Fig. S4).

**Table 3:** Crystallographic parameters and refinement statistics.

	RT^3MB^:DNA	RT^4M^:DNA	RT^4M^:DNA	RT^4M^:DNA
Bound NA/dNTP	*E*-CFCP-TP	*E*-CFCP-TP	ETV-TP	dGTP
PDB code	8XZ1	8X20	8X21	8X22
Data collection				
Beamline	PF BL-17A	PF BL-1A	PF BL-1A	PF BL-1A
Detector	Pilatus3S 6M	Eiger X4M	Eiger X4M	Eiger X4M
Wavelength (Å)	0.98000	1.10000	1.10000	1.10000
Space group	*H*3	*H*3	*H*3	*H*3
Unit-cell parameters (Å)	*a* = *b* = 283.1, *c* = 95.4	*a* = *b* = 284.1, *c* = 95.6	*a* = *b* = 284.4, *c* = 95.7	*a* = *b* = 285.2, *c* = 96.1
Resolution (Å)*	50–2.62 (2.67–2.62)	50–2.70 (2.75–2.70)	50–2.33 (2.37–2.33)	50–2.31 (2.35–2.31)
Unique reflections	85,655	78,960	123,410	127,828
*R*_meas_*^,†^	0.074 (0.896)	0.105 (0.878)	0.157 (0.875)	0.075 (0.926)
Mean *I*/σ (*I*)*	16.8 (2.1)	10.8 (1.7)	7.4 (2.1)	15.0 (2.1)
Completeness (%)*	99.9 (100.0)	100.0 (100.0)	100.0 (100.0)	100.0 (100.0)
Multiplicity*	5.1 (5.2)	5.5 (5.5)	5.5 (5.6)	5.5 (5.6)
Wilson *B*-factor (Å^2^)	61.2	63.3	49.3	48.0
Refinement				
*R*_work_/*R*_free_^‡,§^	0.179/0.223	0.173/0.231	0.176/0.218	0.182/0.219
No. of atoms	17,553	17,537	17,751	17,744
Average* B*-factors (Å^2^)				
All/DNA	75.0/71.9	74.0/70.5	64.0/62.6	65.0/63.1
Ligand	103.4 (*E*-CFCP-TP)	111.2 (*E*-CFCP-TP)	72.2 (ETV-TP)	57.9 (dGTP)
R.m.s.d. from ideal				
Bond lengths (Å)	0.007	0.004	0.012	0.008
Bond angles (°)	0.813	0.805	1.188	0.934
Ramachandran plot^¶^				
Favored	95.8	95.9	96.2	96.7
Outliers (%)	0.3	0.0	0.0	0.0

*Values in parentheses are for the outermost resolution shell.

^†^*R*_meas_ = Σ_h_ Σ_*i*_ |*I*_h,*i*_ −  < *I*_h_ >|/Σ_h_Σ_*i*_* I*_h,*i*_, where < *I*_h_ > is the mean intensity of a set of equivalent reflections.

^‡^*R*_work_ = Σ |*F*_obs_ − *F*_calc_|/Σ *F*_obs_ for 95% of the reflection data used in the refinement. *F*_obs_ and *F*_calc_ are the observed and calculated structure factor amplitudes, respectively.

^§^*R*_free_ is the equivalent of *R*_work_, except that it was calculated for a randomly chosen 5% test set excluded from refinement.

^¶^Ramachandran analysis was performed using MolProbity^50^.

**Figure 3:**
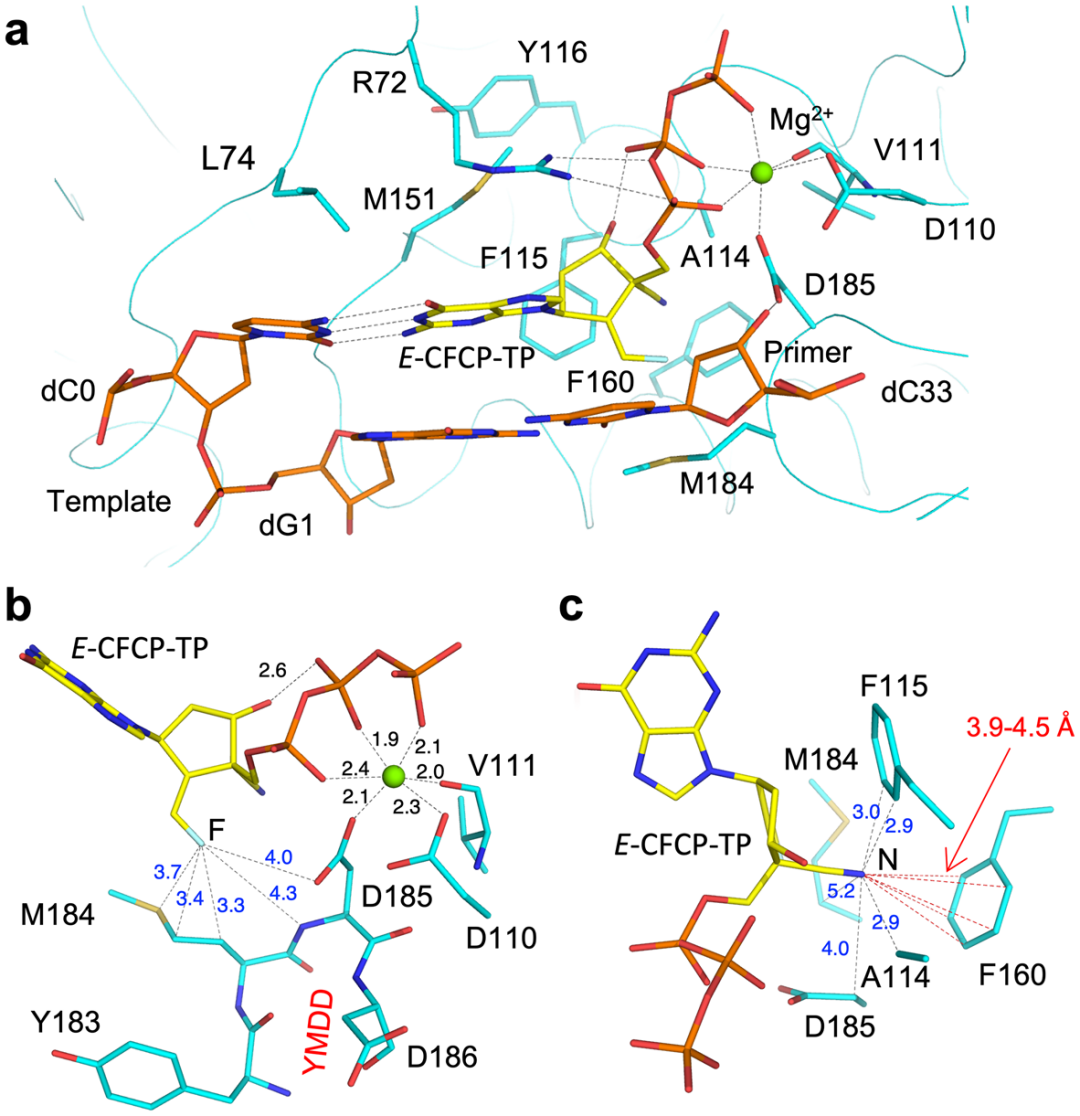
The interatomic interactions between residues creating the N-site and bound *E*-CFCP-TP. (**a**) Overall view for the HIV-1 RT^4M^ N-site with *E*-CFCP-TP. Hydrogen bonds and metal-chelating interactions are indicated by dotted lines. (**b**) Interatomic interactions around fluoromethylene and Mg^2+^. The interactions between the fluorine atom of *E*-CFCP-TP and nearby atoms derived from Met184 and Asp185 are shown by dotted lines, and their distances are also indicated. The YMDD loop is labeled in red. (**c**) Interactions around the 4′-cyano of *E*-CFCP-TP. Interatomic interactions between 4′-cyano nitrogen and nearby atoms derived from the residues creating the hydrophobic pocket (Ala114/Phe115/Phe160/Met184/Asp185). Atomic interactions with residues forming the side wall of the hydrophobic pocket are indicated by black dotted lines, and atomic interactions with Phe160 forming the bottom of the pocket are indicated by red dotted lines. The interatomic distances are also labeled.

**Figure 4:**
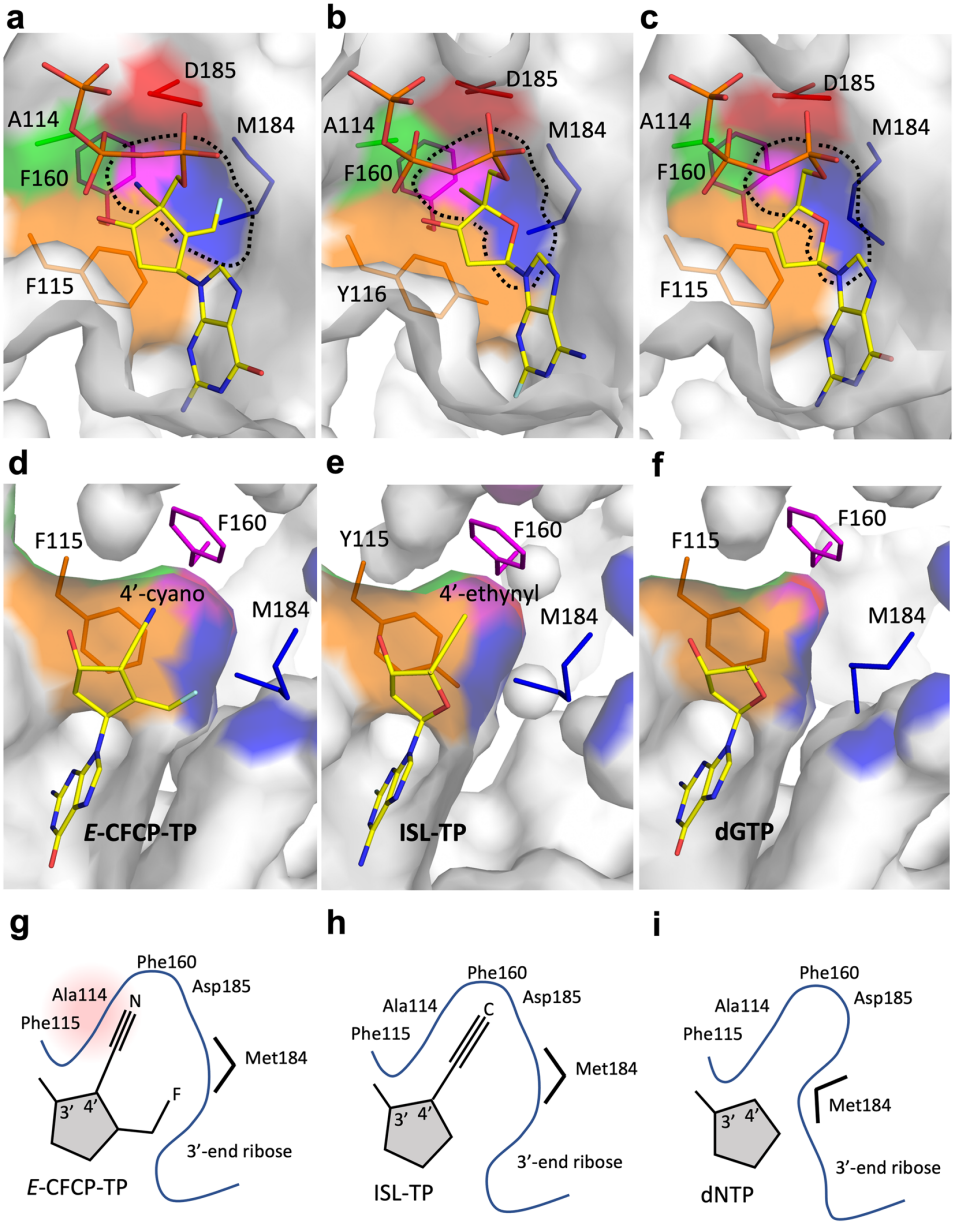
Two distinct states (narrow and expanded state) of the hydrophobic pocket at the N-site in response to bound NAs/dNTP. Surface representation of the hydrophobic pocket with bound *E*-CFCP-TP in RT^4M^ (expanded state) (**a**,**d**), ISL-TP in RT^WT^ (expanded state) (**b**,**e**), and dGTP in RT^4M^ (narrow state) (**c**,**f**). The views from two different directions are shown. The narrow and expanded states of the hydrophobic pocket are primarily due to the conformation of the Met184 sidechain. The residues creating the pocket, i.e., Ala114, Phe/Tyr115, Phe160, Met184, and Asp185, are colored green, orange, magenta, blue, and red, respectively. The schematic representations of the extended and narrow states of the hydrophobic pocket are also shown in (**g–i**). The close contact area is highlighted in light pink.

*E*-CFCP has the same structure as deoxyguanosine except for a 4′-cyano group and exocyclic fluoromethylene. Thus, the interactions between 4′-cyano/fluoromethylene and the amino acids forming the N-site are key to understanding the role of the structural mechanism in the high potency of *E*-CFCP against HBV. The fluorine atom is located midway between the Met184 and Asp185 sidechains with interatomic distances of 3.5–4.0 Å (Fig. 3a,b); the fluoromethylene pushes the Met184 sidechain backward and fills the gap between the Met184 and Asp185 sidechains. The 4′-cyano exploits the deep hydrophobic pocket created by the Ala114, Phe115, Met184, Asp185, and Phe160 sidechains (Fig. 3c). Due to the concavity formed by the Met184 sidechain, the hydrophobic pocket is expanded toward the side of Met184 (Fig. 4); the interatomic distances between Met184 CG and Ala114 CB are 8.0–8.8 Å and 6.0–6.5 Å in the *E*-CFCP-TP and dNTP complexes, respectively (Supplementary Table S1). A similar expansion of the hydrophobic pocket has also been reported in structural studies of HIV-1 RT^WT/1M/3MB^ complexed with ETV-TP^27,28^ and with ISL-TP^32^. ETV-TP has no 4′-modification but has an exocyclic methylene that pushes the Met184 sidechain backward. ISL-TP has no exocyclic projection but has a 4′-ethynyl group only. The backward conformation of the Met184 sidechain in the ISL-TP complex also appears to occur when the 4′-modified group exploits the hydrophobic pocket. Therefore, the expansion of the hydrophobic pocket observed in the present structural study would be strongly enforced by both the exocyclic fluoromethylene and 4′-cyano groups of *E*-CFCP-TP.

The corresponding hydrophobic pocket of HBV RT is created by Phe88, Met204, Ala87, Asp205, and Leu180 and is expected to be shallower due to the presence of Leu180 instead of Phe160^31^. The interatomic distance between the cyano nitrogen and phenyl-ring atoms of Phe160 is 3.9–4.5 Å (Fig. 3c), indicating that the binding position of the cyano group might be well fitted to the shallower hydrophobic cavity of HBV RT. The binding mode of the cyano group is consistent with the fact that the 4′-cyano NAs, such as CdG and CAdA, are highly potent against both HIV-1 and HBV^38^. In contrast, ISL is highly potent only against HIV-1 and less effective against HBV. 4′-ethynyl is longer than 4′-cyano; therefore, a steric clash could occur between the tip of the ethynyl and the Leu180 of HBV RT. Since the 4′-cyano of *E*-CFCP can fit into both the deep and shallow hydrophobic cavities of HIV-1/HBV RT, the weak inhibition activity of *E*-CFCP against HIV-1 might be attributed not to 4′-cyano but fluoromethylene. Notably, the antiviral assay showed that the Q151M mutation alone renders HIV-1 highly susceptible to both ETV and *E*-CFCP. The results strongly suggest that *E*-CFCP could enter the N-site through the transient hydrophobic interactions between fluoromethylene and the Met151 sidechain, a mechanism similar to ETV-TP ingress^22,27^. The Gln151 of HIV-1 RT is located at the entrance to the N-site and may serve as a gatekeeper to exclude ETV/*E*-CFCP with exocyclic methylene/fluoromethylene from entering.

### Deviated binding mode of the cyclopentyl moiety of *E*-CFCP-TP attenuates drug-resistant F160M/M184V mutations

**Figure 5:**
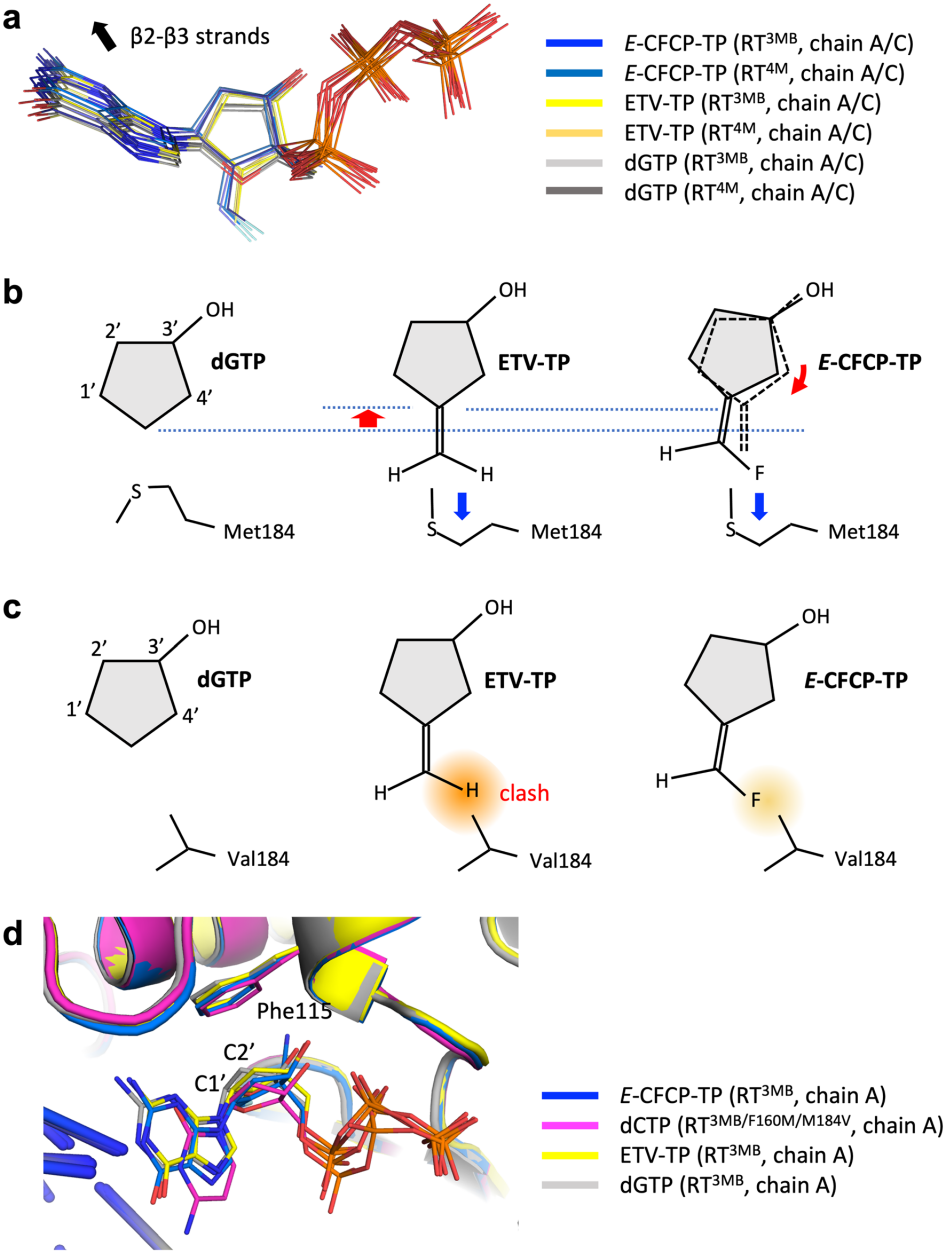
Deviated binding of *E*-CFCP-TP. (**a**) Superimposition of bound* E*-CFCP-TP, ETV-TP, and dGTP reported in this study. The location of β2–β3 strands is indicated by the arrow. (**b**) Schematic diagram showing the binding position and orientation of the five-membered ring moiety of *E*-CFCP-TP, ETV-TP, and dGTP. Deviated binding of *E*-CFCP-TP is indicated by red arrows. The pushing of Met184 by methylene/fluoromethylene is indicated by blue arrows. (**c**) Structural effect of drug-resistant M184V mutation on exocyclic methylene (ETV) and fluoromethylene (*E*-CFCP-TP). A severe steric clash is expected to occur between the methylene of ETV and the Val184 sidechain, while the fluorine of *E*-CFCP is expected to be located relatively distant from the Val184 sidechain. (**d**) Deviated binding mode of bound dCTP in RT^3MB/M184V/F160M^^22^ and *E*-CFCP-TP in RT^3MB^ colored blue and magenta, respectively. Bound ETV-TP and dGTP in HIV-1RT^3MB^ are also shown in yellow and gray, respectively. As shown here, the C1′ and C2′ of the bound dCTP occupy the same position as those of *E*-CFCP-TP.

Superimposition of the bound *E*-CFCP-TP, ETV-TP, and dGTP in HIV-1 RT^3MB/4M^ highlights the deviated binding of the cyclopentyl moiety of *E*-CFCP-TP (Fig. 5); the cyclopentyl moiety is shifted approximately 0.6 Å toward Met151 and skewed by approximately 10°. Such a deviated* E*-CFCP-TP binding mode has not been previously reported in any HIV-1 RT:DNA:NA/dNTP complexes. Apparently, the deviated binding observed in the present study resulted from the avoidance of a significant steric clash between fluorine and the Met184 sidechain (Fig. 5). This deviated binding affects the orientation of the 4′-cyano group, which protrudes long from the cyclopentyl ring, i.e., the 4′-cyano is inserted at an angle into the hydrophobic pocket, resulting in unfavorable close interatomic contact between cyano nitrogen and the Ala114/Phe115 sidechain atoms with distances of 2.9–3.2 Å (Figs. 3c, 4). Such close contact is energetically unfavorable, which might be related to the fact that the bound *E*-CFCP-TP is perturbed with relatively higher *B*-factor values in the N-site as described above. In contrast, the 4′-ethynyl of ISL-TP is located at the center of the hydrophobic pocket, suggesting that ISL-TP perfectly fits into the expanded hydrophobic pocket without any close contact. It should also be noted that the present deviated binding mode of *E*-CFCP-TP involved the slight movement of its guanine base moiety toward β2–β3 strands (Fig. 5a), and this movement is likely compatible with the substitution of smaller valine (I63V/L74V) in the β2–β3 region. However, primer extension assays demonstrated that HIV-1 RT^4M (3MB+L74V)^ had a lower susceptibility to *E*-CFCP-TP compared to RT^3MB^ (Supplementary Fig. S3). These results suggest that the presence of the L74V mutation alone does not provide sufficient compatibility for binding with *E*-CFCP-TP. Additionally, a cell-based assay revealed that HIV-1^5MB (3MB+I63V/L74V)^ exhibited the highest susceptibility to *E*-CFCP (Table 2), indicating that both I63V/L74V mutations may be required for a tight binding of *E*-CFCP-TP to the N-site.

The bulky *E*-CFCP-TP does not perfectly conform to the N-site but rather expands its hydrophobic pocket with 4′-cyano and fluoromethylene. Furthermore, *E*-CFCP-TP itself slightly deviates to fit into the expanded N-site structure. Importantly, the C1′ and C2′ positions of the bound *E*-CFCP-TP correspond exactly to those of the bound dCTP deviating into the N-site of drug-resistant HIV-1 RT^3MB/F160M/M184V^ (Fig. 5d)^22^. The N-site of RT^3MB/F160M/M184V^ has a small protrusion due to the CG of Val184 and a Phe115 bulge caused by F160M substitution, which might be structural factors causing drug resistance. The deviated binding position of *E*-CFCP-TP is apparently less affected by these structural properties caused by F160M/M184V mutations. Therefore, this deviated binding mode of *E*-CFCP-TP could be a structural basis for the high antiviral activity of *E*-CFCP against HIV-1^3MB^/HBV harboring the drug-resistant mutations F160M/M184V (L180M/M204V in HBV RT).

## Conclusion

This study demonstrated that HIV-1 with the HBV-associated 3MB (Y115F/F116Y/Q151M) mutation in RT turned to be highly susceptible to *E*-CFCP as well as ETV. In addition, HIV-1^3MB^ with drug-resistant mutations F160M/M184V was found to be resistant to ETV but remained highly susceptible to *E*-CFCP. These findings are in line with the known fact that *E*-CFCP exhibits high antiviral activity against both wild-type and drug-resistant HBV. To gain further insight, we determined the X-ray structure of RT^3MB^ and RT^4M^, which serve as surrogates for HBV RT, in complex with *E*-CFCP-TP. The structures revealed that the cyclopentene moiety of the bound *E*-CFCP-TP is slightly skewed and deviated, wherein the position partly corresponds to that of the bound dNTP observed in the HIV-1 RT^3MB^ with drug-resistant mutations F160M/M184V. Thus, they evade the structural effects caused by the F160M/M184V substitutions. The structures reported in this study provide novel insights into the interactions between the RT N-site and NAs/dNTP. They can also serve as a benchmark for further molecular dynamics and docking simulations aimed at identifying new NAs that are more potent and do not allow drug-resistant mutations.

## Methods

### Virus preparation, replication kinetics, and antiviral assays

The HIV-1 variants used in this study were prepared as previously described^27^. Briefly, the genes encoding HIV-1 RT p51 and p66 used in this study originated from the HIV-1 clone NL4-3 (GenBank: M19921.2). All site-specific mutations were introduced by inverse PCR, and infectious HIV-1 clones with mutations in RT were constructed using an IN-Fusion HD Cloning Kit (Clontech, Mountain View, CA, USA). Infectious HIV-1 variants were obtained by transfecting HEK293T cells.

For the virus replication assay, MT-4 cells were exposed to each HIV-1 variant and cultured for 7 days. The amount of the HIV-1 p24 antigen in supernatants was measured on days 0, 1, 3, 5, and 7 using a Lumipulse G1200 system (Fujirebio, Tokyo, Japan). All assays were performed in duplicate. The anti-HIV-1 activity of antiviral compounds was examined by performing p24 assays, as described above. The potency of HIV-1 inhibition by a compound was determined based on the production of p24 antigens compared with that of drug-free controls. All assays were conducted in duplicates of at least two independent experiments. *E*-CFCP was synthesized as previously described^39^. *E*-CFCP-TP was synthesized by TriLink BioTechnologies Inc. (San Diego, CA, USA), and ETV-TP was synthesized as previously described^27^. Other antiviral NAs were obtained commercially.

### RT enzyme assay

The enzyme activity of HIV-1 RT harboring HBV-associated amino acid mutations was determined using a SYBR Green-based real-time PCR-enhanced RT (SG-PERT) assay as previously described^40^ with minor modifications. Briefly, the reaction mixture (20 μL) used contained 1 ng/mL of purified recombinant RT (as described below/elsewhere), 4U RNase inhibitor (TaKaRa Bio Inc., Shiga, Japan), primers (400 nM each, as shown below), 0.1 μL pre-heated (65 °C, 5 min) MS2 RNA (Roche, Basel, Switzerland), and 10 µL PowerUp SYBR Green Master Mix (Applied Biosystems, Waltham, MA, USA). The reaction was conducted under the following conditions: 30 min at 37 °C for RT reaction, 5 min to activate Taq polymerase, and 40 cycles of amplification: 5 s at 95 °C and 30 s at 60 °C. The primers used included FWD: 5′-TCC TGC TCA ACT TCC TGT CGA G-3′, REV: 5′-CAC AGG TCA AAC CTC CTA GGA ATG-3′. The RT enzyme activity of each variant was compared with that of the wild-type using the ΔΔCt method. All assays were performed in triplicate.

### Primer extension assay

To determine the drug susceptibility of each RT variant, primer extension assays were performed as described in a previous report^41^, with minor modifications. In brief, the DNA template (T_d31_: 5′-CCA TAG CTA GCA TTG GTG CTC GAA CAG TGA C-3′) was annealed to a 5′-Cy3-linked P_d18_ primer (5′-Cy3-GTC ACT GTT CGA GCA CCA-3′) in a 1:1 molar ratio. The template/primer hybrid (100 nM), RT^WT/3MB/4M^ (50 nM),* E*-CFCP-TP (0.0, 0.1, 1.0, 10, 100 µM), and a dNTP mixture (100 nM) were incubated in a buffer containing 50 mM Tris–HCl pH 8.0, 50 mM NaCl, and 10 mM MgCl_2_ at 37 °C for 30 min. The RT products were then resolved under denatured conditions on a 10% polyacrylamide 7M urea gel, the fluorescence was measured, and the data were analyzed using iBright 1500FL (ThermoFisher Scientific, Waltham, MA, USA). The IC_50_ values of *E*-CFCP-TP were determined as 100% extension for the no-drug control.

### Recombinant expression and purification of HIV-1 RT mutants

Overexpression and purification of HIV-1 RT^3MB^ and RT^4M^ were performed according to a previously described method^27^. Briefly, the expression of HIV-1 RT p66 (with 3MB/4M mutations) and p51 subunits was induced in *Escherichia coli* BL21-CodonPlus(DE3)-RIL (Agilent Technologies, Santa Clara, CA, USA) with pET28_His_6_-p51 and pCDF_p66 by 0.1 mM isopropyl-β-D-thiogalactopyranoside at 37 °C for 4 h. The matured p66-p51 heterodimer was purified from the crude extract using Ni-affinity and ion-exchange chromatography. Thereafter, the purified HIV-1 RT^3MB/4M^ heterodimer was dialyzed against a buffer containing 10 mM Tris–HCl pH 8.0, 100 mM NaCl, and 1 mM dithiothreitol. Next, the protein concentration was assessed using a Bradford protein assay kit (BioRad Laboratories, Hercules, CA, USA) with bovine serum albumin as a standard. The RT activity for recombinant HIV-1 RT^3MB/4M^ was confirmed using a reverse transcriptase assay kit (Roche, Basel, Switzerland) according to the manufacturer’s instructions. The sample was concentrated to ~ 20 mg/mL using an Amicon Ultra centrifugal filtration device with a 50-kDa cut-off (Merck Millipore, Darmstadt, Germany).

### HIV-1 RT^3MB/4M^:DNA complex preparation and gel-filtration chromatography

The hairpin 38-mer DNA aptamer mimicking template-primer DNA was used to crystallize the RT^3MB/4M^:DNA complex^28,42^. The DNA aptamer was purchased from Hokkaido System Science, Co., Ltd (Hokkaido, Japan). The 100-μM DNA aptamer in a buffer containing 10 mM Tris–HCl pH 8.0 and 1.0 mM EDTA was heated at 80 °C for 10 min and then gradually cooled to 4 °C. The annealed DNA aptamer and purified RT mutants were mixed at a molar ratio of approximately 1.5:1.0, and the mixture was loaded onto a HiLoad 16/600 Superdex 200 pg gel-filtration column (Cytiva, Marlborough, MA, USA) with a loading buffer containing 10 mM Tris–HCl pH 8.0 and 50 mM NaCl to separate the RT:DNA complex from the p51 soluble aggregate and surplus unbound DNA aptamer. Each fraction was analyzed by SDS PAGE and native PAGE to verify subunit content and purity. The fractions for peak elution were concentrated separately to ~ 20 mg/mL, and each sample was used for the crystallization experiments.

### Crystallization and X-ray diffraction data collection

The RT^3MB/4M^:DNA was crystallized using the hanging-drop vapor diffusion method at 20 °C in a 24-well Linbro plate. The crystallization droplet was prepared by mixing 0.5 μL RT^3MB/4M^:DNA sample and 0.5 μL reservoir solution and equilibrated with 500 μL reservoir solution. The crystals appeared 48 h from setup and grew to a maximum size of 0.5 × 0.5 × 0.2 mm, using a reservoir solution containing 20 mM Bis-Tris–HCl pH 6.0, 20–60 mM di-ammonium hydrogen citrate, 20 mM MgCl_2_, 2–4.5% PEG 6000, 0–2.4% sucrose, and 4.8–6.0% glycerol. The crystal was picked up in a nylon loop and transferred to a cryoprotectant solution containing 20 mM Bis-Tris–HCl pH 6.0, 50 mM NaCl, 20 mM MgCl_2_, 60 mM di-ammonium hydrogen citrate, 12% PEG 6000, 4.8% sucrose, 25.6% glycerol, and 2.5 mM *E*-CFCP-TP/ETV-TP/dGTP. After soaking for ~ 5 min, the crystal was again picked up and flash-cooled in liquid nitrogen at 100 K. All X-ray diffraction data were collected with synchrotron radiation at 100 K at the Photon Factory (PF) BL-1A and BL-17A (Tsukuba, Japan). The raw X-ray diffraction data were processed using the XDS^43^ and AIMLESS programs in the CCP4 suite^44^.

### Structure determination and model refinement

The structure was determined using the molecular replacement method with the previously reported HIV-1 RT^3MB^:DNA:dGTP complex as a search model (PDB code, 6KDN)^28^. Model refinement was performed using REFMAC5^45,46^, BUSTER^47^, and Phenix^48^, and model fitting was performed manually using Coot^49^. TLS parameters were introduced in the final stage of model refinement. To verify the validity of the atomic model of bound *E*-CFCP-TP/ETV-TP/dGTP, a simulated annealing mFo–DFc omit map was generated using BUSTER^47^. The quality of the final refined model was assessed using MolProbity^50^. Crystallographic parameters and refinement statistics are summarized in Table 3. All molecular drawings were generated using PyMOL ver. 2.3.4 (Schrödinger LLC, New York, NY, USA).

### Supplementary Information

Supplementary Information.

## Data Availability

The atomic coordinates and structure factor amplitudes of the HIV-1 RT^3MB^:DNA:*E*-CFCP-TP, RT^4M^:DNA:*E*-CFCP-TP, RT^4M^:DNA:ETV-TP, and RT^4M^:DNA:dGTP have been deposited in the RCSB Protein Data Bank (http://www.rcsb.org) under accession codes 8X1Z, 8X20, 8X21, and 8X22, respectively. Other data generated and/or analyzed during this study are available from the corresponding authors upon reasonable request.
