# Supplementary material for: Deviated binding of anti-HBV nucleoside analog E-CFCP-TP to the reverse transcriptase active site attenuates the effect of drug-resistant mutations
Source: Sci Rep. 2024 Jul 8;14:15742. doi: 10.1038/s41598-024-66505-z (PMC11231328; doi:10.1038/s41598-024-66505-z)
Supplement: Supplementary file 1 — Supplementary Information. [file 41598_2024_66505_MOESM1_ESM.pdf]

Supplementary information

Deviated binding of anti-HBV nucleoside analog *E*-CFCP-TP to the reverse transcriptase active site attenuates the effect of drug-resistant mutations

Yoshiaki Yasutake[1,2,*], Shin-ichiro Hattori[3], Hiroki Kumamoto[4], Noriko Tamura[1], Kenji Maeda[3,5], Hiroaki Mitsuya[3,6,7,*]

[1]Bioproduction Research Institute, National Institute of Advanced Industrial Science and Technology (AIST), Sapporo 062-8517, Japan.
[2]Computational Bio Big-Data Open Innovation Laboratory (CBBD-OIL), AIST, Tokyo 169-8555, Japan.
[3]National Center for Global Health and Medicine (NCGM) Research Institute, Tokyo 162-8655, Japan.
[4]Department of Pharmaceutical Sciences, Nihon Pharmaceutical University, Saitama 362-0806, Japan.
[5]Division of Antiviral Therapy, Joint Research Center for Human Retrovirus Infection, Kagoshima University, Kagoshima 890-8544, Japan.
[6]Experimental Retrovirology Section, HIV and AIDS Malignancy Branch, National Cancer Institute, National Institutes of Health, Bethesda, MD 20892, USA.
[7]Department of Clinical Sciences, Kumamoto University Hospital, Kumamoto 860-8556, Japan.

*Corresponding authors. Yoshiaki Yasutake: Tel, +81-11-857-8514; Fax, +81-11-857-8980; E-mail, y-yasutake@aist.go.jp. Hiroaki Mitsuya: Tel, +81-33-202-7181; Fax, +81-33-202-7364; E-mail, hmitsuya@hosp.ncgm.go.jp.

Supplementary Table S1. Comparison of interatomic distances between Met184 CG and Ala114 CB for the structures reported in this and previous studies.

	Atoms	Distances (Å)		PDB code	References
		Chain A	Chain C		
RT^{3MB}:DNA:*E*-CFCP-TP	Met184 CG – Ala114 CB	8.24	8.01	8X1Z	This study
RT^{4M}:DNA:*E*-CFCP-TP		8.71	8.32	8X20	This study
RT^{3MB}:DNA:ETV-TP		8.46	8.22	6KDM	[28]
RT^{4M}:DNA:ETV-TP		8.44	8.23	8X21	This study
RT^{3MB}:DNA:dGTP		6.23	6.14	6KDN	[28]
RT^{4M}:DNA:dGTP		6.29	6.22	8X22	This study
RT^{WT}:DNA:dCTP		6.04	-	6UIT	[36]
RT^{WT}:DNA:dATP		6.45	6.39	5TXL	[37]
RT^{WT}:DNA:ISL-TP		8.02	-	5J2M	[32]

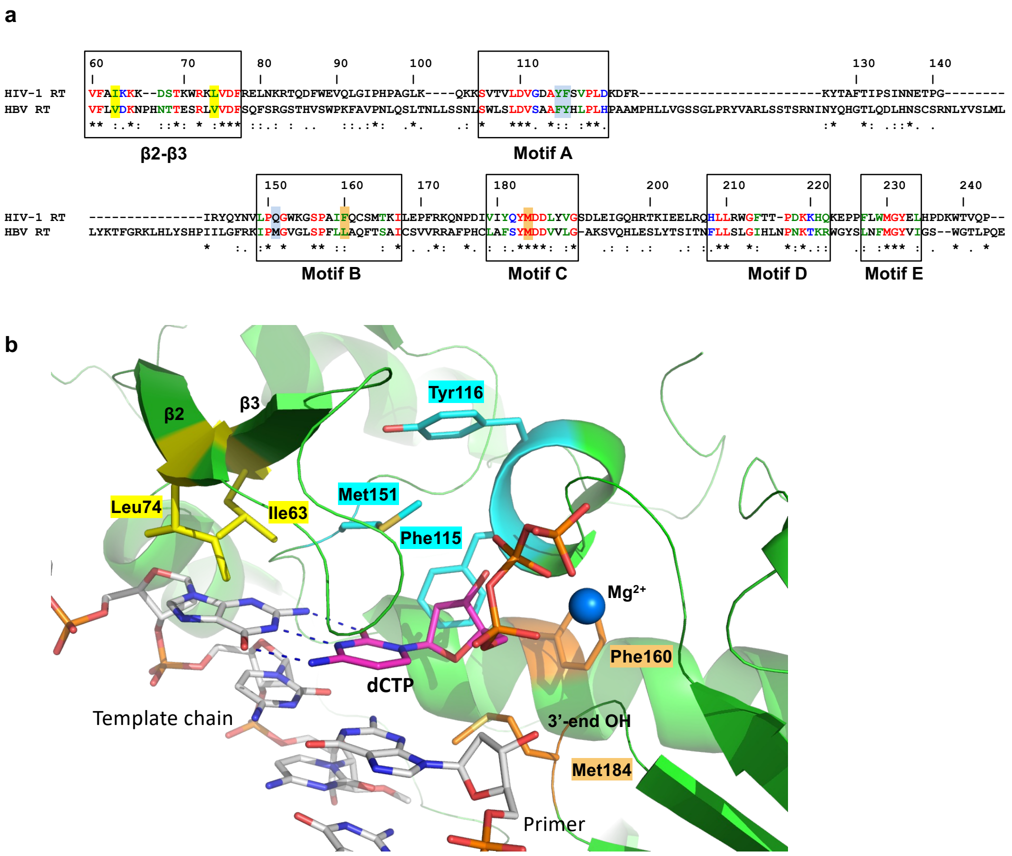

Supplementary Figure S1. Design of HIV-1 mimicking the HBV RT N-site. (**a**) Amino acid sequence alignment between HIV-1 and HBV RT. The conserved motifs (motifs A–E and β2–β3) are boxed. Identical and strongly similar residues in the motifs are colored red and green, respectively. 3MB mutations (Y115F/F116Y/Q151M) and additional I63V/L74V in β2–β3 are highlighted in blue and yellow, respectively. The drug resistance-related residues M184/F160 are highlighted in orange. The identical, strongly similar, and weakly similar residues are colored red, green, and blue, respectively. (**b**) HBV-mimicking HIV-1 RT N-site structure. The figure was prepared using the previously reported RT^{3MB}:DNA:dCTP complex (PDB code, 6KDK) [28]. The residues for 3MB (Y115F/F116Y/Q151M) and I63/L74 are indicated by cyan and yellow, respectively. M184/F160 is shown in orange.

a

b

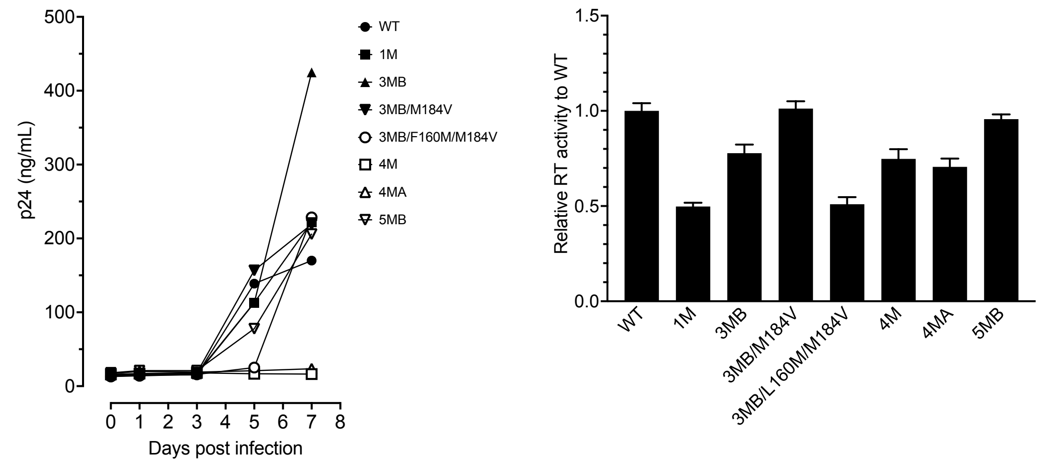

Supplementary Figure S2. Impact of HBV-associated amino acid residues in the RT of HIV-1. (**a**) Viral replication kinetics of HIV-1 mutants were determined. HIV-1 mutants, except 4M and 4MA, moderately lost their replication capacity compared with that of HIV-1 WT. HIV-1 4M and 4MA were apparently unable to propagate. Assays were performed in duplicate, and plots represent mean values. (**b**) Enzyme activity of HIV-1 RTs harboring HBV-associated and drug-resistant mutations was measured and compared with that of wild-type HIV-1 RT (WT). HIV-1 $RT^{3MB/M184V}$ and RT^{5MB} retained their RT enzyme activity, whereas other mutants lost up to half of their enzyme activity. Assays were performed in triplicate, and data represent mean ± SD.

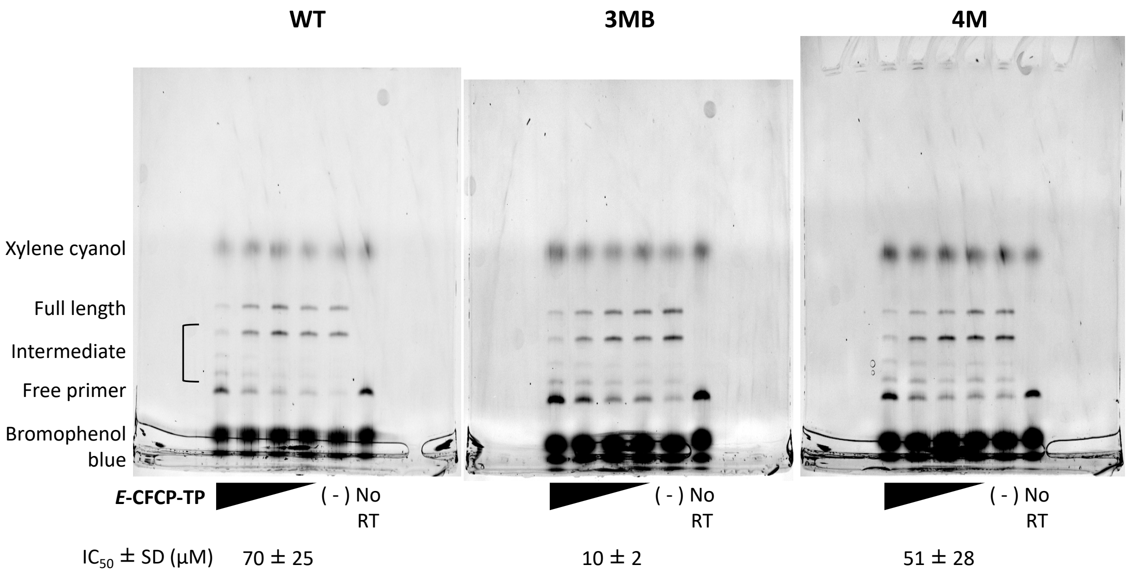

Supplementary Figure S3. Susceptibility changes of each RT variant (RT^{WT}, RT^{3MB}, and RT^{4M}) against *E*-CFCP-TP. Primer extension reactions were performed using HIV-1 RT^{WT} (WT), RT^{3MB} (3MB), and RT^{4M} (4M) in the presence of fixed concentrations of dNTPs, T_{d31}/P_{d18}, and $MgCl_2$, with the addition of 100, 10, 1, and 0.1 μM of *E*-CFCP-TP, or without *E*-CFCP-TP (-). The rightmost lane has no RT added as background. The full-length elongation products were measured. The IC_{50} values for *E*-CFCP-TP were determined using % reduction from the no-drug control and mean ± SD obtained from three independent experiments. Represent images are shown.

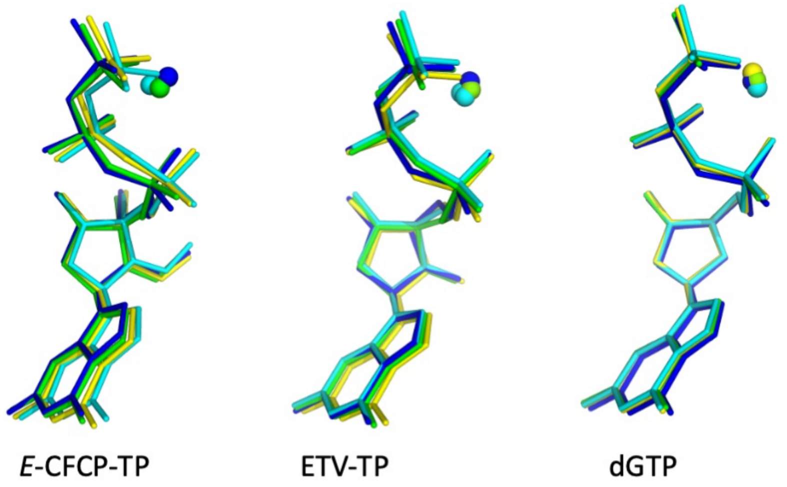

R.m.s.d. range for pairs of each chain (Å)

	E-CFCP-TP	ETV-TP	dGTP
E-CFCP-TP	0.38-0.48	0.42-0.87	0.39-0.95
ETV-TP	-	0.21-0.33	0.39-0.60
dGTP	-	-	0.22-0.30

Supplementary Figure S4. Superimposition of bound *E*-CFCP-TP/ETV-TP/dGTP in HIV-1 RT^{3MB} and RT^{4M}. Cyan, RT^{3MB} chain A; Green, RT^{3MB} chain C; Yellow, RT^{4M} chain A; blue, RT^{4M} chain C. RMSD value ranges for pairs of each chain are also listed. As shown here, *E*-CFCP-TP is more perturbed than ETV-TP and dGTP within the N-site of $RT^{3MB/4M}$.
